# Improving prokaryotic transposable elements identification using a combination of *de novo* and profile HMM methods

**DOI:** 10.1186/1471-2164-14-700

**Published:** 2013-10-11

**Authors:** Choumouss Kamoun, Thibaut Payen, Aurélie Hua-Van, Jonathan Filée

**Affiliations:** 1 Laboratoire Evolution, Génomes, Spéciation, CNRS UPR9034/Université Paris-Sud, Gif-sur-Yvette, France

**Keywords:** Insertion Sequence (IS), Miniature Inverted repeat Transposable element (MITE), Data mining, Genome and metagenome, Profile HMM

## Abstract

**Background:**

Insertion Sequences (ISs) and their non-autonomous derivatives (MITEs) are important components of prokaryotic genomes inducing duplication, deletion, rearrangement or lateral gene transfers. Although ISs and MITEs are relatively simple and basic genetic elements, their detection remains a difficult task due to their remarkable sequence diversity. With the advent of high-throughput genome and metagenome sequencing technologies, the development of fast, reliable and sensitive methods of ISs and MITEs detection become an important challenge. So far, almost all studies dealing with prokaryotic transposons have used classical BLAST-based detection methods against reference libraries. Here we introduce alternative methods of detection either taking advantages of the structural properties of the elements (*de novo* methods) or using an additional library-based method using profile HMM searches.

**Results:**

In this study, we have developed three different work flows dedicated to ISs and MITEs detection: the first two use *de novo* methods detecting either repeated sequences or presence of Inverted Repeats; the third one use 28 in-house transposase alignment profiles with HMM search methods. We have compared the respective performances of each method using a reference dataset of 30 archaeal and 30 bacterial genomes in addition to simulated and real metagenomes. Compared to a BLAST-based method using ISFinder as library, *de novo* methods significantly improve ISs and MITEs detection. For example, in the 30 archaeal genomes, we discovered 30 new elements (+20%) in addition to the 141 multi-copies elements already detected by the BLAST approach. Many of the new elements correspond to ISs belonging to unknown or highly divergent families. The total number of MITEs has even doubled with the discovery of elements displaying very limited sequence similarities with their respective autonomous partners (mainly in the Inverted Repeats of the elements). Concerning metagenomes, with the exception of short reads data (<300 bp) for which both techniques seem equally limited, profile HMM searches considerably ameliorate the detection of transposase encoding genes (up to +50%) generating low level of false positives compare to BLAST-based methods.

**Conclusion:**

Compared to classical BLAST-based methods, the sensitivity of *de nov*o and profile HMM methods developed in this study allow a better and more reliable detection of transposons in prokaryotic genomes and metagenomes. We believed that future studies implying ISs and MITEs identification in genomic data should combine at least one *de novo* and one library-based method, with optimal results obtained by running the two de novo methods in addition to a library-based search. For metagenomic data, profile HMM search should be favored, a BLAST-based step is only useful to the final annotation into groups and families.

## Background

Insertion Sequences (ISs) and their non autonomous derivatives known as Miniature Inverted repeat Transposable Elements (MITEs) are the simplest kinds of prokaryotic mobile DNA. ISs are small DNA segments ranging from 1 to 3.5 kb, generally encoding a transposase that catalyses the mobility of the elements. Most ISs are surrounded by terminal inverted repeats (IRs) and flanked by direct repeats (DRs). MITEs are thought to be originated from internal deletion of complete ISs and generally lack any recognizable open reading frames (ORFs). They use the transposases encoded by the corresponding full elements in *trans* for their mobility [1]*.*

ISs and MITEs abundance in prokaryotic genomes is highly variable [2] but they generally occupy a substantial fraction, up to 40% in the *Orientia tsutsugamushi* genome [3] with an average of 1 to 10% in Bacteria [4] and Archaea [5]. In addition, metagenomic analyses revealed that transposases are the most abundant and ubiquitous genes in nature [6]. The quantitative importance of ISs is coupled with a large diversity of families and mechanisms of transposition. Based on transposase sequence similarities, ISs have been classified in 25 different families that belong to three main classes of enzymes: the DDE transposase, the Serine Recombinase and the Tyrosine Recombinase [4]. Consequently, ISs are considered as major players of genome evolution and plasticity, mediating gene transfers and promoting genome duplication, deletion and rearrangement [7]. Due to their abundance and diversity, ISs and MITEs identification and annotation have represented a longstanding challenge, partially solved with the availability of a reference database that compile a large body of ISs (ISFinder at https://www-is.biotoul.fr/). Thus, several studies have used the referenced sequences in the ISFinder database to mine various collections of genomic data using BLAST softwares (see for example [2,5,8-10]). However, with the development of high-throughput sequencing techniques leading to the availability of thousands complete genomes and metagenomes, ISs and MITEs identification and annotation require more sophisticated and integrated approaches. Recently, ISSaga have been then developed to automate IS annotation in complete genomes [11]. ISSaga used a relatively simple library-based methods using BLAST seeded with the ISFinder sequences and classify them into families. Although ISSaga have represented a significant progress in the field, the efficiency of library-based approaches in identifying transposable elements is questionable for two reasons. First, the efficiency of library based method is critically dependent on the quality and the exhaustiveness of the database used. Several families such as IS4 for example display extremely elevated levels of divergence, with many emerging clusters that show very weak level of sequence conservation with the other members of the family [12]. Other families such as IS91 for example show a very low sampling effort with only twenty sequences present in ISFinder, mainly in a single bacterial clade (alpha Proteobacteria). Second, library-based methods are unable to identify new families that display no similarities with existing families. This limit is especially problematic with MITEs that do not encode for a transposase and that display low level of similarities with autonomous ISs [13]. For this reason, in Eukaryotes more than 50 different methods have been developed to identify and annotate transposable elements [14]. These methods could be divided in library based method and *de novo* methods. *De novo* methods do not need a set of reference sequences to works: they used various approaches relying on the structural properties of the transposable elements as the presence of terminal repeats or the fact that transposons are generally duplicated in multiple copies in a given genome. However it’s striking that *de novo* methods have been underused to mine prokaryotic genomes. Thus, the goal of this study was to develop alternative and more elaborate methods than BLAST-based approaches to improve ISs and MITEs identification. We build three new pipelines, two using *de novo* methods (searching for repeated sequences and searching for the presence of IRs) and the third one using an alternative library-based method with profile Hidden Markov Models (HMM) searches.

We tested these different pipelines against different datasets:

– A genomic dataset of 30 archaeal genomes previously annotated by us for ISs and MITEs with a BLAST-based method [5]. Our results demonstrate that *de novo* methods increased significantly ISs (+10%) and MITEs (+50%) identification compared to library based methods. The gain was important with highly divergent families such as IS4 or IS200/605 and for MITEs that display only very weak similarities with previously identified ISs. In addition to be fast, reliable for non-specialist users and generating low level of false positive, these methods offer the advantage to produce outputs with complete IS sequences (transposase, accessory genes, IRs…) directly usable for databases as ISFinders. Similar results were obtained using 30 additional bacterial genomes annotated in the ISFinder database.

– We also tested some marine metagenomic datasets including samples composed of 1 kb reads and 250 bp reads. Our results show that HMM searches outclass a BLAST-based approach, finding many more transposase genes (up to +50%) and generating a considerable lower level of false positive compared to BLAST. Thus, we suggested that BLAST based methods should be avoided to detect transposase on metagenomic data and should be reserved to the final annotation step in order to class the elements by IS families. Moreover future studies on ISs and MITEs may combine at least one *de novo* method of detection in addition to a library based approach.

## Methods

### Overview

In order to improve ISs and MITEs identification, we have constructed three different work-flows: two *de novo* pipelines that search for repeats sequences and for Inverted Repeats (IRs) and a library-based pipeline using HMM alignment profile searches. These methods were then benchmarked using genomic and metagenomic datasets.

### De novo pipelines

**Figure 1:**
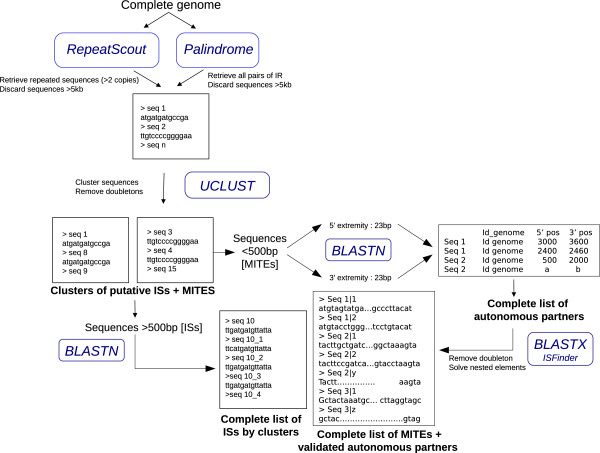
**Simplified workflow of the *de novo *methods.** Running softwares are indicated in blue, rectangles schematize the different output files at each step.

**Figure 2:**
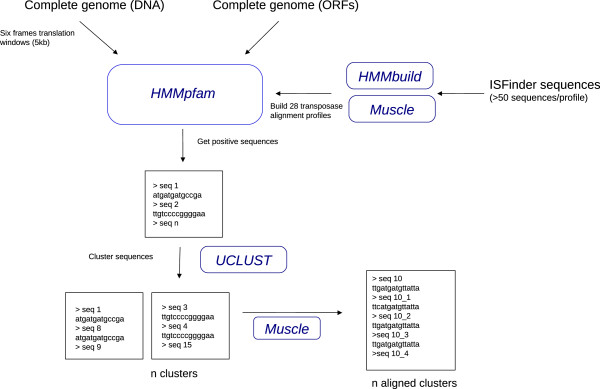
**Simplified workflow of the profile HMM search.** Running softwares are indicated in blue, rectangles schematize the different output files at each step.

Our *de novo* pipelines used two different approaches to detect ISs and MITEs (Figures 1 and 2). The first one called “Repeats search” (Figure 1) used the RepeatScout algorithm [15]. RepeatScout detects repeated sequences in a given genome and generates a consensus of these sequences (with default parameter and word size l = 9). The second path called “IRs search” (Figure 2) used the Palindrome software of the EMBOSS package [16] which identifies the IRs in the input sequences (with the following parameters: minpallen = 10, maxpallen = 50, gaplimit = 2000, nummismatches = 2). All sequences delimited by IR pairs are then extracted. In order to avoid doubleton, sequences bordered with IRs but present less than 2 times were removed. The consensus sequences generated by RepeatScout and the sequences delimited by IR following Palindrome were then clustered using UCLUST with default parameters [17]. At this stage, we have a list of clusters of putative MITEs and ISs. For the putative ISs (sequences larger than 500 bp) we compiled exhaustively all the related sequences (including truncated copies) using a BLASTN (E value = 10e-5) [18] against the genome. For the MITE candidates (sequences smaller than 500 bp) we need to identify the autonomous partners to be sure that these sequences are true transposable elements. We acquired the terminal 23 bp at each end of the sequence (which correspond roughly to the IRs of the elements) and used this sequence to BLASTN (E value = 10e-1) the genome. We obtained a list of sequences that are present between the two matching terminal 23 bp. These sequences were filtered to be smaller than 3 kb and doubletons were removed. Finally the nested elements (elements inserted in each other as Russian dolls) are separated and reconstructed separately. These potential partners were then blasted (BLASTX with E value = 10e-5) against the ISFinder database (04/2011 update) to be certain that these sequences are homologous to *bona fide* ISs. At the end of the process, we obtain a list of files with all the putative ISs and a file containing all the MITEs with the corresponding autonomous partners.

### Profile HMM pipeline

The “profile HMM search” pipeline used in entry files either the complete genomes (nucleotides) or the list of ORFs (amino acids). In the first case, as profile search methods proceed with protein sequences only, the genome is then cut in 5 kb segment and translated using a six frames window. Transposase sequences are searched using the hmmpfam software of the HMMER2 [19] package using 28 transposase sequence profiles (with default parameters and E value = 10e-5). These profiles were built as follow: for each family, all the sequences present in the ISFinder database have been retrieved according to their family name. For underrepresented families in ISFinder as IS91 for example, additional homologous sequences were retrieved using BLASTP (E value = 10e-5) searches against a NR database maintained locally and regularly updated (minimum number of sequences per profiles = 50). For large families with little similarities between subgroups we have generated several different profiles (example: IS4). These sets of sequences are aligned with MUSCLE [20], alignments are refined manually using the Jalview sequence editor [21] and the profiles are then constructed using the hmmbuild software [19]. Sensitivity of the profiles has been improved using the calibration software hmmcalibrate. The sequences encoding a putative transposase identified using these profiles are extracted and clustered using the UCLUST [17] softwares and the sequence clusters are finally aligned with MUSCLE [20]. At the end of the pipeline we will have a list of files containing the different clusters of related sequences encoding a putative transposase.

### Test with prokaryotic genomes

The benchmarking of our different methods has been tested against a set of 30 Archaeal genomes previously annotated by us for ISs and MITEs [5]. In this previous study, ISs have been identified using a BLASTP-based approache against ISFinder and MITEs have been identified using BLASTN with the IS sequences previously identified (E value = 10e-5). All of these annotated transposons and their respective coordinates in the genomes were downloaded from the ISFinder database and compared with those identified with our pipelines. Additionally, we have randomly chosen 30 bacterial genomes available in the ISFinder database that cover the major bacterial phyla (Cyanobacteria, Firmicute, *Deinococus*-*Thermus* groups etc…) and compared the performance of our pipelines with the ISFinder annotations.

### Test with metagenomic datasets

We have tested the HMM pipeline against a simulated metagenomic dataset and a real set of metagenomic sequences. The simulated datasets correspond to the genome of the archaeon *Sulfolobus solfataricus* that has been fragmented in 1000 bp, 250 bp and 100 bp sequences to resemble of typical metagenomic reads generated by Sanger or NGS methods. We have chosen the genome of *Sulfolobus solfataricus* because it offers the advantage to carry a very large panel of ISs (149 copies) in addition to a large diversity of sequences (27 elements belonging to 12 IS families). The real dataset have been chosen randomly from the CAMERA database at http://camera.calit2.net: (i) the Pacific Beach Sand metagenome composed of 4981 non-redundant sequences for a total of approximately 6 Mb (average size of the reads: 1 kb), (ii) the marine SAR11 bacterial clade metagenome composed of 10300 sequences for a total of approximately 2.5 Mb (average size of the reads: 250 bp) and (iii) the Sargasso sea Metagenome JCVI_SMPL_1103283000007 with 10 000 sequences for a total of 9.2 Mb (average size of the reads: 900 bp). Performance of the HMM pipeline were compared with the results obtained with a BLASTX against the ISFinder sequences (04/2011 update) with *E* value = 10e-5. Each alignment was checked manually to avoid false positives and ambiguous cases were blasted against a NR database to identify homologous sequences (BLASTX with *E* = 10e-5).

### Technical details

Pipelines were written either in Perl (version 5.14.2) or Python (version 2.7.3). Computation described in this article were performed on a UNIX server [64 Intel(R) Xeon(R) CPU 2.13 GHz with 8 GB of RAM]. The full *de novo* pipelines analysis for the 30 genomes was done in approximately 3 hours, where as the HMM pipeline, with the 28 transposase alignment profiles were performed in approximately 100 hours. The calculation time for the metagenome dataset using the HMM profiles scales linearly with the number of sequences, in average 40 minutes per 1000 reads.

### Scripts are available at

– Profile HMM search: http://www.legs.cnrs-gif.fr/Realisations/Tmp/HMMSearch_web.tar.gz

– IRs search: http://www.legs.cnrs-gif.fr/Realisations/Tmp/IR_search.tar.gz

– Repeats search: http://www.legs.cnrs-gif.fr/Realisations/Tmp/Repeat_search.tar.gz

Newly identified ISs have been deposited on the ISFinder database.

## Results and Discussion

### Pipelines validation: test with 30 Archaeal and 30 bacterial genomes

We constructed three different work-flows: two *de novo* pipelines that search for repeats sequences (called “Repeats search”) and for Inverted Repeats (called “IRs search”) and a library-based pipeline using HMM alignment profile searches. These methods were then tested using diverse genomic and metagenomic datasets. We first validated the performance of our different pipelines by detecting the presence of 190 different ISs and 26 different MITEs previously identified by BLAST in a set of 30 Archaeal genomes [5]. The respective performances of the different methods are given in Table 1. The performances of the HMM pipeline fed with 28 transposase alignment profiles with the BLAST searches against ISFinder sequences are globally comparable. Three known single copies have been lost during the HMM search process but 5 new multi-copies ISs have been identified. By contrast, our *de novo* pipelines improve significantly ISs and MITEs annotation. Repeats search increased notably the number of multi-copy ISs compared to BLAST (10 new ISs, + 9%) and clearly outclasses the latter concerning MITEs identification (13 new MITEs, +50%). However, Repeats search generates a significant level of false positives that correspond to various other kind of mobile elements such as group-II Intron encoding a reverse transcriptase or conserved genes in prophage as integrase, in addition to several duplicate genes as rDNA 16S for example. Nevertheless as these false positive hits encoded for highly conserved and generally well annotated genes, they could be easily filtered with a simple BLASTX against a NR database at the end of the process. Alternatively, an increase of the copy number repeats threshold to 4 or 5 instead of 3 removed most of these false hits but it will also decrease significantly the sensitivity of the pipeline. Conversely, IRs searches give no false positives but seem less powerful to detect ISs (-48% compared to BLAST). This is directly related to the fact that several IS families as IS607, IS605, IS91 and IS110 do not displays terminal IR. If we excluded these families, the IR searches give comparable results to the Repeats search with the detection of 3 additional new ISs. In fact, symmetrically with the Repeats search, the IRs search turn out to be especially efficient to detect MITEs with an increase of about 50% compared to BLAST. Taken together, combination of our *de novo* pipelines allows us to detect 15 new ISs (+8%) and 18 new MITEs (+70%) compared to the BLAST-based approach (see the next sections for a complete description of these new elements).

**Table 1:** Respective performance of the different methods against reference dataset of 30 Archaeal genomes

	**BLAST search (E < 10e-5)**	**HMM search (p < 10e-5)**	**Repeats search**	**IRs search**
Number of different ISs	190	187	125	67
Number of different ISs > 2 copies	115	120 (+4,3%)	125 (+8.7%)	67 (-47,8%)
Number of different MITEs	26	26 (+0%)	39 (+50%)	39(+50%)
False positives	0	11	99	0

Additionally, we have also tested a sample of 30 bacterial genomes annotated in the ISFinder database (Additional file 1: Table S1). Performances of the different pipelines are remarkably similar with those obtained with Archaeal genomes. The best performances are provided by the Repeats search pipeline (with 8 additional new elements found) and the HMM pipelines. The IR search give even better results with the bacterial dataset (-16,5%) compared with the Archaeal datasets (-48%). This result is mainly due to the weaker proportion of IS families that do not displayed IRs in the bacterial dataset as IS605 and IS607 families. Finally, it should be noted that our *de novo* pipelines detected 21 potential MITEs in the bacterial genomes (with a total of 189 different ISs), a proportion roughly comparable with the Archaeal genomes (44 MITEs for 250 ISs).

Finally, *de novo* methods provide output files directly usable for transposon databases *ie* providing the complete transposon sequence, not only the transposase sequences (or the recombinase). Finding the complete sequence (IRs, the additional ORFs etc…) of a transposon detected on the basis of its transposase may appears trivial, but this task is in fact time-consuming and not easy to automate. Eye examination of the local alignment sequence bordering the transposase is generally required and the ISSaga software developed to find transposases do not provide options to find the complete corresponding IS sequences. By providing simple and fast methods to find the complete sequence of the transposon, *de novo* methods implemented in this study offer sensitive and reliable ways to find the complete sequence of the transposon. Nevertheless, *de novo* methods are unable to identify single copy ISs which represent a significant fraction of the total diversity of IS in prokaryotic genomes (Table 2). This limitation is inherent to the *de novo* methodologies which filter the candidate sequences on the basis of their repetitions. Consequently, an exhaustive study of IS needs optimally the combination of library-based and *de novo* methods. In a long term, by feeding the sequence libraries with the recursive use of *de novo* methods, we can expect to cover a sufficient sequence space to solely used similarity-searches methods. However, it seems quite evident that our present knowledge of the true diversity of transposons in the prokaryotic world is highly incomplete. Thus, we recommend that future studies on ISs and MITEs may combine *de novo* and library-based methods, ideally IRs search, Repeats search and profile HMM search.

**Table 2:** Performance of a BLAST based search compared to a profile HMM search for diverse simulate and real metagenomes

**Dataset**	**BLAST ISs false positives**	**HMM search ISs false positives**
*S. solfataricus*, 1 kb	149 0	133 0
*S. solfataricus*, 250 bp	149 0	129 0
*S. solfataricus*, 100 bp	149 0	29 0
*SAR11* Metagenome, ~250 bp	20 NA	13 NA
PBS Metagenome, ~1 kb	189 281	264 7
JCVI Metagenome, ~900 bp	44 114	87 0

*Sulfolobus* datasets correspond to *in silico* fragmented genomes and the results indicated the number of individual transposases identified. For the real marine metagenomes, the results indicate the number of apparent true transposases identified (false or ambiguous positives have been removed after careful alignments visualization) in addition to the numbers of false positives. NA: not applicable (see the text for further details).

### Pipelines validation: test with simulated and real metagenomes

Metagenomic data differ notably from traditional genomic data by the short size of the reads that severely complicate gene annotation [22]. We have compared the respective performance of the HMM-search pipelines compared to a BLAST-based method with the ISFinder sequences. We tested four different metagenomes: three simulated metagenomes consisting of the genome of *Sulfolobus solfataricus* fragmented in 1 kb, 250 bp and 100 bp segments, and three marine metagenomes composed of 1 kb, 900 bp and 200 bp reads. For the latter, we have carefully checked the sequence alignments to avoid false (or ambiguous) positives to be sure that the identified ISs are true elements.

Concerning the fragmented *Sulfolobus* genome, the HMM search pipeline leaded to the identification of 133, 129 copies and 29 out of 149 (respectively 89%, 87% and 20%) identified with BLAST (Table 2). It should be noted that ISFinder database already contains all the *Sulfolobus* ISs, consequently the result obtained by BLAST correspond to an optimum. Thus it’s not surprising that the profile HMM searches do not fit exactly with the BLAST approches. In fact, all the transposases missed by the profile HMM pipeline with 1 kb and 250 bp belong to a single group of IS5 elements (IS*C1212* and IS*C1236* elements) which show only very weak similarities to other IS5 elements and also vary significantly among themselves. Moreover, the spacing of the DDE catalytic residues does not align with that of other IS5 family members [5] and this would probably explain the difficulties to identify them based of alignment profiles search methods. Finally, due to the lack of sequence similarities with very short input sequences, the performance of the HMM pipelines with 100 bp fragments decreases strongly.

Concerning the “real” metagenomic datasets, the results vary according to the size of the reads (Table 2). With the 1 kb reads (Pacific Sand PBM and Sargasso Sea JCVI) the efficiency of the HMM pipeline is considerably better than BLAST (+ 40 and +51% respectively). In addition, BLAST tends to generate a lot of false positives displaying very short aligned regions (<150nt) (Table 2). For example, with the JCVI sample, the number of false positives corresponds to 114 hits for 44 apparent “true” ISs. To reduce the level of false positives, it’s possible to decrease of the BLAST *E* values threshold to have a more stringent criterion of similarities. This option dramatically reduced the numbers of hits: for example, with *E* value = 10e-10 instead of 10e-5, the number of ISs identified in the JCVI sample is divided by a factor 5. At the opposite, the HMM search gives a very low level of false positives (0 for the JVCI sample and 7 for the PBS sample). With HMM searches, some metagenomic sequences also lead to multiple matches with two or three different profiles. This phenomenon is frequent with DDE families with overlapping sequence spaces, as observed with the elements matching with the IS481 profile in addition to the IS3 and/or IS630 profiles. This may complicate the eventual annotation of the corresponding sequences and it seems useful to combine the HMM search (detection step) with a BLAST search against ISFinder to annotate by families the previously identified sequences.

Concerning the sample composed of short reads as the SAR11 clade metagenome (250 bp in average), the efficiencies of both methods seem equally limited (Table 2) and it seems obvious that many elements have been missed compared to the results obtained with the 1 kb sequence samples. In addition, the short sizes of the aligned sequences complicate seriously the identification and the removal of the false positives. In conclusion, the sensitivity of the HMM searches makes this method more successful than BLAST to identify ISs on metagenomic samples, generating less false positives and providing a better picture of the IS diversity. The only limitation seems to be the size of the reads, short sequences (<300 bp) appearing too small to provide enough sequence information to identify ISs.

### Description of the new ISs found with the de novo methods

Utilization of the two *de novo* pipelines with the 30 Archaeal genomes allowed us to discover 15 new ISs (Table 3). A majority of these ISs does not belong to known families or displays little similarities with existing ones (ex: IS*Ape1*, IS*Fac11*, IS*Mac26* etc…). This would explain why library-based methods failed to identify them. These elements display typical transposon structure: duplication in multiple copies, presence of IRs at the extremities and sometimes generation of target site duplication (DRs). As all these elements have closed homologs in diverse bacterial and archaeal genomes, they could belong to previously unidentified emerging families. We also observed an apparent overrepresentation of members of the IS200/IS605 family: four of them display enough sequence conservation to be associated with this family. Absence of identification of these elements during the initial study with a BLAST-based approach in 2007 is more puzzling. The IS200/IS605 is a loosely defined family that is clearly polyphyletic: it clustered together Serine Recombinase type and Tyrosine Recombinase type enzymes. These elements have been clustered together because they may share a second and non-essential ORF of unknown function [4]. Until recently, this family has received little attention, but during the past few years many studies concerning its diversity, their mechanism of transposition and their roles on genome evolution were published [23-25]. Many new sequences was probably identified and added to libraries, thus it’s then possible that the sequence sampling in the ISFinder database increases enough to allow identification of more distant homologs in our archaeal genomes. Additionally, we cannot rule out that these elements were missed as there is an important mass of IS200/IS605 elements present in these Archaeal genomes (36 distinct elements for a total of 210 copies). This situation well illustrates the fact that library based approaches critically depend on the quality and on the diversity of the sequence sampling in the database, representing a major weakness compare to *de novo* methods.

**Table 3:** **Main characteristics of the new ISs identified using *de novo *methods**

**Host**	**IS name**	**Family**	**Copy number C P**	**ISFinder similarity**	**DR**	**IR**
*M. hungateii*		ISCNY (?)	9 3	10e-12 IS*Plu15*	0	0
*A. pernix*		?	8 2	-	0	0
*S. tokodaii*		IS200/605	4 0	10e-20 IS*Bce3*	0	0
*F. acidarmanus*		?	4 0	-	6	27
*F. acidarmanus*		IS200/605	4 0	6e-36 IS*Dge19*	0	0
*F. acidarmanus*		?	6 1	-	9	17
*F. acidarmanus*		IS3	4 1	5e-29 IS*Bce13*	0	16
*F. acidarmanus*		IS4 (?)	3 0	5e-9 IS*Mbov2*	0	34
*T. volcanium*		IS200/605	5 0	5e-18 IS*Tsib1*	0	0
*M. acetivorans*		?	13 6	-	0	24
*M. acetivorans*		?	2 1	-	0	16
*M. acetivorans*		?	2 0	-	0	21
*M. acetivorans*		?	5 0	-	0	19
*N. pharaonis*		IS200/605	3 0	8e-35 IS*Clte2*	0	10
*T. kodakarensis*		?	2 1	-	0	14

The host genomes and the names according the ISFinder nomenclature is indicated, the IS family when it was defined, the copy number (C: complete, P: Partial), the similarity with ISFinder (04/2011 update) and the size of the IRs and DRs.

Finally, it should be noted that we have identified with *de novo* methods 13 additional IS-like elements that display typical transposon structure (presence of IRs, multiple copies…). These IS-like elements show any sequence similarities outside their corresponding genomes. So far, the sequence sampling is not sufficient to identify catalytic motives as the DDE for example to be certain that these elements are *bona fine* transposable elements. It should be noted that several similar “orphans”ISs have already been reported in Archaea, mainly in Halophilic and Methanogens species [5]. Thus, we can hypothesize that these 13 elements are true and additional new transposons belonging to rare and unknown IS families. Increasing availability of new genomes and metagenomes will definitively validate (or not) this assumption.

### Description of the new MITEs found with de novo methods

**Figure 3:**
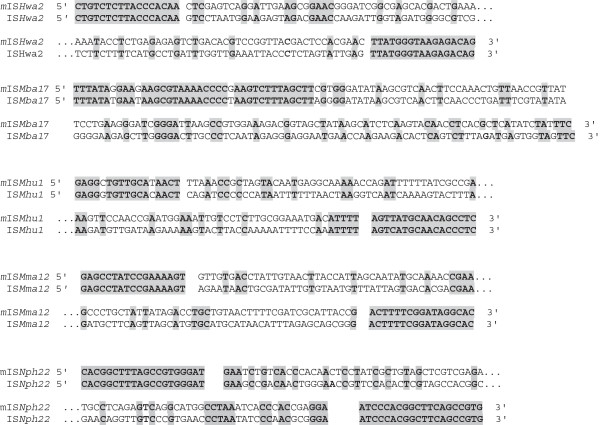
**Sequence alignment of 3′ and 5′ ends of a representative set of MITEs identified in this study with their putative autonomous IS partners.** Names of MITEs and their autonomous ISs are given according the ISFinder nomenclature, gray colors indicate conserved residues and the spaces delimitate the IRs of each elements.

Due to the lack of any ORFs, MITEs are difficult to detect with library-based approaches. Nevertheless, 26 MITEs families have been evidenced in the 30 archaeal genomes studied previously [5]. These elements have been detected on the basis of their overall sequence similarities with autonomous ISs, using a simple BLASTN against each genome seeded with the complete sequence of the ISs previously identified. With the implementation of *de novo* methods, we have now identified 18 new MITEs (Table 4). Unlike the new autonomous ISs identified in this study, there is no apparent bias towards peculiar families: the new MITEs derive from ISs that belong to 9 different families, without over representation of unknown or poorly defined families as IS200/605 for example. These new MITEs are often present with quite high copy numbers (>10), displaying typical MITE structures: presence of IRs that are similar with those of autonomous ISs, and sometimes generation of DRs. All but one are associated with previously identified ISs in our earlier study [5]. The only exception is a MITE in the *Thermoccocus kodakarensis* genome which is associated with an IS discovered in this study (IS*Tko3).* This observation raises the question: why so many MITEs have not been detected using BLAST-based methods? Alignments of a representative set of MITE sequences with their putative autonomous IS partners (Figure 3) show that the level of similarities between them is very low. In fact, only the borders corresponding to the IRs are conserved which correspond generally to segments shorter than 20nt (including some mismatches). Even for the IS200/IS605 elements, if the 5′ ends seem to be more conserved, the 3′ ends display virtually no or very weak similarities ( for example IS*Mba17* in Figure 3). This would explain that a BLASTN seeded with the sequence of the complete ISs failed to be exhaustive and missed more than 50% of the MITEs in our 30 archaeal genomes. In addition, we identified 19 other “MITE-like” structures appearing as repeated elements displaying IRs and sometimes DRs. However, we failed to identify any autonomous IS partner associated with them. There are two likely and not exclusive explanations for this observation:

– These elements are associated with an IS that have been lost during the course of the evolution.

– They are associated with unknown ISs, showing no similarities with recognized IS families.

**Table 4:** **Main characteristics of the new MITEs identified using *de novo *methods**

**Host**	**Putative IS**	**Family**	**Size**	**Copy number**	**IR**	**DR**
*M. hungatei*	IS*Hmu1*	?	136	6	15	4
*T. kodakarensis*	IS*Tko3*	?	274	5	14	0
*H walsbyi*	IS*Hma7*	IS*200/605*	368	17	0	0
*H walsbyi*	IS*Hwa2*	IS*4*	237	34	17	5
*H walsbyi*	IS*Hma12*	IS*200/605*	570	13	0	0
*H walsbyi*	IS*Hwa6*	IS*1595*	165	7	25	8
*M. burtonii*	Is*Mbu10*	IS*5*	185	5	16	0
*M. barkeri*	IS*Mba19*	IS*5*	147	5	15	0
*M. barkeri*	IS*Mba11*	IS*1634*	247	23	20	6
*M. mazei*	IS*Mba17*	IS*200*/*605*	418	5	0	0
*M. mazei*	IS*Mma9*	IS*630*	89	5	16	0
*M. acetivorans*	IS*Mac10*	IS*1634*	249	15	18	0
*M. acetivorans*	IS*Mac2*	IS*1182*	160	14	26	4
*M. acetivorans*	IS*Mma12*	IS*5*	140	24	16	3
*M. acetivorans*	IS*Mac21*	IS*L3*	179	6	22	7
*M. acetivorans*	IS*Mac15*	IS*5*	252	12	17	0
*M. acetivorans*	IS*Mac2*	IS*1182*	169	7	25	0
*N. pharaonis*	IS*Nph22*	IS*200/605*	341	2	0	0

The host genomes, the potential IS autonomous partner and its family when defined are indicated. Lenght (in bp), copy number and size of the DRs and IRs are also mentioned.

Thus, the apparent difficulties to identify the autonomous partner due to the high level of sequence divergence between them indicated that we underestimated the number of MITEs present in the archaeal genomes.

## Conclusion

In this work, we have developed three new alternative methods to improve ISs and MITEs identification in prokaryotic genomes. Compared to a routinely used BLAST-based approach, *de novo* methods based on repeats detection and identification of the IRs improve notably the detection of ISs and MITEs. These methods have led to the identification of more than 30 new transposons (+20%) in a reference dataset of 30 archaeal genomes. *De novo* methods appear powerful to detect ISs belonging to poorly defined families, highly divergent ones or emerging groups with no or few representatives in the sequence libraries. As MITEs display in prokaryotes very few sequence similarities with their autonomous IS partners (mainly in the IRs), the advantages of *de novo* methods are magnified. We also developed an alternative library-based method to detect transposases using profile HMM searches. Tested against metagenomic samples, this method supplants a classic BLAST-based method, increasing the number of putative transposases (up to +50%) and generating less false positives. These results are in favor of a generalization of *de novo* methods in data mining for prokaryotic transposons, ideally combining a library-based method in addition to a *de novo* method. A better efficiency would be reach using both *de novo* methods (IRs search and repeats search) in addition to a BLAST or a profile HMM search. Concerning metagenomes, data mining for transposase using classical BLAST-based methods should be replaced by profile HMM searches; BLAST should be used only in a second step for the annotation and the classification into families.

## Abbreviations

IS: Insertion sequence; MITEs: Miniature inverted repeat transposable elements; IR: Inverted tepeat; DR: Direct repeat; HMM: Hidden markov model.

## Competing interests

The authors declare that they have no competing interests.

## Authors’ contribution

JF conceived the study, performed data analysis and wrote the manuscript. Implementation of the different work-flows in Perl and Python was done by JF, CK, TP and AHV. All authors have read and approved the final manuscript.

## Supplementary Material

Additional file 1: Table S1 Respective performances of the different methods against an ISFinder dataset of 30 bacterial genomes. For each genome, the numbers correspond to the ISs identified in the ISFinder database and using our different pipelines. The number of MITEs identified using our *de novo* methods are also indicated. Click here for file
