# Supplementary material for: Improving prokaryotic transposable elements identification using a combination of de novo and profile HMM methods
Source: BMC Genomics. 2013 Oct 11;14:700. doi: 10.1186/1471-2164-14-700 (PMC3852290; doi:10.1186/1471-2164-14-700)
Supplement: Additional file 1: Table S1 — Respective performances of the different methods against an ISFinder dataset of 30 bacterial genomes. For each genome, the numbers correspond to the ISs identified in the ISFinder database and using our different pipelines. The number of MITEs identified using our de novo methods are also indicated. [file 1471-2164-14-700-S1.pdf]

	ISFinder	ISFinder >2copies	HMM Search	Repeats Search	IR Search	MITES
Stenotrophomonas maltophilia K279a	14	12	14	12	7	2
Thermus thermophilus HB8	2	2	2	2	2	1
Rhodospirillum rubrum ATCC 11170	4	0	4	0	0	
Bacillus coagulans 36D1	8	4	8	5	5	
Methylocella silvestris BL2	4	0	4	0	0	
Herminiimonas arsenicoxydans	6	1	6	1	0	
Helicobacter pylori J99	1	0	0	0	0	
Fusobacterium nucleatum subsp. nucleatum ATCC 25586	8	5	7	5	3	
Deinococcus geothermalis DSM 11300	10	6	9	6	5	
Parabacteroides sp. D13	2	0	2	0	0	1
Beggiatoa sp. PS	3	0	2	0	0	
Clostridium thermocellum ATCC 27405	17	10	16	10	7	
Bacillus halodurans C-125	16	11	16	11	7	1
Candidatus Desulforudis audaxviator MP104C	3	2	3	3	3	1
Akkermansia muciniphila ATCC BAA-835	1	0	1	0	0	
Anaeromyxobacter dehalogenans 2C	4	3	4	4	3	
Synechocystis sp. PCC6803	7	5	7	5	5	
Anaeromyxobacter sp. Fw109-5	6	1	5	2	1	
Anaeromyxobacter sp. K	8	3	8	3	2	
Arthrobacter aurescens TC1	1	1	1	1	1	
Arthrobacter chlorophenolicus A6	1	0	1	1	0	1
Arthrobacter sp. FB24	4	1	4	1	0	
Bacillus cereus ATCC 10987	4	0	4	0	0	
Bacteroides thetaiotaomicron VPI-5482	6	4	6	5	4	3
Delta proteobacterium MLMS-1	7	3	8	4	4	4
Geobacillus kaustophilus HTA426	5	5	8	6	6	3
Pelodictyon phaeoclathratiforme BU-1	5	5	5	5	5	
Cyanothece sp. PCC 7425	7	5	5	6	5	1
Aliivibrio salmonicida LFI1238	11	6	10	6	5	1
Psychromonas ingrahamii 37	5	2	5	2	1	2
**Total**	180	97	175	106	81	21
